# Consideration of Acute Porphyria in an Emergency Department Patient: A Case Report and Discussion of Common Pitfalls

**DOI:** 10.5811/cpcem.2022.9.57507

**Published:** 2022-11-07

**Authors:** Anthony Rios, Lisa Kehrberg, Hillary E. Davis

**Affiliations:** * University of Tennessee, Department of Biochemistry, Knoxville, Tennessee; † University of Tennessee Medical Center, Department of Emergency Medicine, Knoxville, Tennessee; ‡ Family Medicine, Lincolnshire, Illinois

**Keywords:** acute hepatic porphyria, porphobilinogen, aminolevulinic acid, recurring abdominal pain, case report

## Abstract

**Introduction:**

Porphyria refers to a group of disorders associated with defects in heme synthesis. They can be associated with severely debilitating features, including abdominal pain, psychiatric symptoms, neurological defects, and cardiovascular irregularities. Although these diseases are rare, patients with attacks often do present to the emergency department (ED) where consideration of porphyria is generally not included in the differential.

**Case Report:**

Here, we examine a case of a 16-year-old male who presented to our ED for evaluation of recurring abdominal pain and auditory hallucinations in which porphyria was considered by the emergency physician.

**Discussion:**

Not considering acute porphyria in patients with recurring neurovisceral symptoms in the ED can lead to missed opportunities for diagnosing such pathologies.

## INTRODUCTION

Porphyria refers to a group of rare metabolic disorders caused by deficiencies in crucial enzymes in the biosynthesis of heme resulting in the accumulation of toxic precursors, delta-aminolevulinic acid (ALA) and porphobilinogen (PBG).^1^ These disorders can be broken down into two types clinically: acute (neurovisceral) or non-acute (cutaneous).^2^ The acute types of porphyria include acute intermittent, hereditary coproporphyria, variegate, and ALA-dehydratase deficient. Acute porphyria can present with severe but non-specific symptoms including abdominal pain, neurological changes, psychiatric disturbances, and cardiovascular irregularities. In addition, acute porphyria attacks can be triggered by medications, alcohol, infection, endogenous hormones, illicit drugs, low caloric intake, or stress that require an increased demand for heme.^1^

Patients with porphyria who present to the emergency departments (ED) with acute attacks are often misdiagnosed. Emergency physicians do not naturally consider porphyria in differential diagnoses given their unfamiliarity with the disorder and the non-specific and variable symptoms with which these patients present. Further, on the rare occasion that the disorder is considered, emergency physicians are often unfamiliar with ordering and obtaining the correct screening tests.

Here we present a case in which the emergency physician did consider the possibility of acute porphyria in a patient with recurring neurovisceral symptoms. We identify and discuss several pitfalls in the hope that these patients can be more readily identified and treated in the acute care setting.

## CASE REPORT

A 16-year-old Black male presented to our ED at a 710-bed academic institution with two days of auditory hallucinations and epigastric abdominal pain. The patient had a history of psychosis and had required an inpatient psychiatric hospitalization for both auditory and visual hallucinations at the age of 12. He did not experience hallucinations at baseline and was doing well in school. The patient’s family had a significant history of mental illness: the patient’s uncle at the time was institutionalized in a psychiatric hospital, and the patient’s brother, later diagnosed with schizophrenia, had presented similarly with auditory hallucinations at the age of 13. For the prior two years, the patient had repeated episodes of upper abdominal pain that were associated with nausea and vomiting. He was followed by a pediatric gastroenterologist and had undergone both a colonoscopy and esophagogastroduodenoscopy just three months prior with no remarkable findings. On review of external charts, we learned he had presented to varying EDs on 12 separate occasions for abdominal pain and had been prescribed multiple antibiotics for presumed colitis on several occasions.

Upon arrival, the patient was afebrile (36.6°C) with a heart rate of 81 beats per minute, respiratory rate of 18 breaths per minute, blood pressure of 128/83 millimeters of mercury (mm Hg), and an oxygen saturation of 99% on room air. Physical exam was notable for a soft, non-distended abdomen with epigastric pain that was not replicated by palpation. He had no focal neurological deficits. He appeared anxious and tearful but was organized in thought. Although he reported auditory hallucinations, he did not actively seem to be responding to internal stimuli.

Screening tests typical for psychiatric patients at our ED were ordered, including a nine-panel urine drug screen, a complete blood count, serum chemistries, liver function tests, an ethanol level, and a urinalysis. The ethanol level was not elevated, and the urine drug screen was unremarkable. An elevated total bilirubin of 2.0 milligrams per deciliter (mg/dL) (reference range: 0.3–1.0 mg/dL) was detected while all other lab values were within the normal range. The patient was subsequently placed in a monitored psychiatric room and referred out to inpatient psychiatric facilities.

After 24 hours, the patient was evaluated by a different emergency physician. Given the patient’s continued abdominal pain, an abdominal ultrasound was ordered, which demonstrated no sonographic abnormality. After reviewing the patient’s medical history and being concerned about the possibility of porphyria, the physician ordered a spot urine porphyria assay. No amber or light-sensitive collection tubes were available in the hospital; consequently, the sample was collected in a test tube wrapped in foil. The hospital laboratory subsequently sent the specimen to an outside facility for analysis. The patient was sent to an inpatient psychiatric facility the following day.

The urine porphyria assay results were available five days after initial collection and were as follows: urine uroporphyrins of 24 micrograms per deciliter (mcg/dL) (reference range: 0–2.0 mcg/dL), urine pentacarboylporphyrins of 5 mcg/dL (0–2.0 mcg/dL), urine heptacaroxylporphyrins of 8 mcg/dL (0–2.0 mcg/dL), urine coproporphyrin I of 46 mcg/dL (0–2.0 mcg/dL), and urine coproporphyrin III of 134 mcg/dL (0–2.0 mcg/dL). Given the notable elevations, the emergency physician contacted the patient’s primary care physician for further follow-up, as the phones of the patient’s family had been disconnected. On review of external records, it was found that the patient had three more ED visits for abdominal pain during the following four months.

**CPC-EM Capsule**

What do we already know about this clinical entity?

*Porphyria attacks cause a high degree of morbidity but are under-recognized in emergency departments secondary to lack of understanding of the diseases and their associated confirmatory tests*.

What makes this presentation of disease reportable?

*We consider a rare case in which the physician was concerned about porphyria in an emergency department patient but unfortunately ordered the wrong test secondary to lack of familiarity*.

What is the major learning point?

*When a clinical suspicion arises for porphyria, initiating the workup in the emergency department is ideal. Physicians need to be cognizant of the timing and types of confirmatory diagnostics*.

How might this improve emergency medicine practice?

*More patients with porphyria may be identified leading to a subsequent decrease in repetitive and costly generalized workups and better porphyria patient outcomes*.

## DISCUSSION

Initiating a clinical suspicion for porphyria is difficult as the condition may present with broad and non-specific symptoms. Patients with porphyria often undergo expensive and broad workups prior to their ultimate diagnosis.^3^ Further, when there is a concern for the disease, the diagnosis may be elusive secondary to clinicians’ lack of familiarity with confirmatory tests, and the timing of the tests themselves.

In the case we describe, porphyria was appropriately included in the differential diagnosis; however, the incorrect screening test was ultimately obtained. In acute exacerbations, the levels of porphyrin precursors generally increase. These levels can either normalize or remain elevated in an asymptomatic state.^4^ Here, a urine porphyrin assay was attained as opposed to the precursor assay: the urine porphobilinogen (PBG). During acute porphyria attacks, a substantial elevation in PBG or ALA, a porphyrin precursor that becomes PBG in the second step of heme biosynthesis, occurs.^5^ Urine porphyrins, alternatively, are produced in the later stages of the heme biosynthesis pathway. These are non-specific for porphyria disorders; they are elevated in acute exacerbations but also in common hepatocellular and biliary pathologies, oral contraceptive use, and alcohol consumption.^6^–^8^ Urine porphyrins aid in the classification of porphyria, but they are not used alone to diagnose the disorder. However, given that urine porphyrins may persist longer between attacks, they can increase the sensitivity of testing and should be collected simultaneously.

Ordering the correct test is not straightforward because not only is the precursor molecule desired, but there are also several assays to measure urine PBG along with different aliases. Some of the most common include the following: “PBG quantitative,” “porphobilinogen,” “PBG trace kit,” “Watson-Schwartz test,” and the “Hoesch test.”^4^ Samples can be collected either by “spot” (single-occurrence) or 24-hour collection, with the latter being slightly more sensitive. Recently, experts have advocated for obtaining only spot samples as 24-hour collections can delay necessary therapeutic measures.^4^ Given the time constraints of patient interactions and limited available resources, spot collections lend themselves to being more feasible in acute care settings.

Spot collections should be obtained from preferably symptomatic patients using freshly voided urine, while excluding first-voided morning samples, samples after significant fluid intake, and samples voided after 8 pm.^9^ At least one milliliter is necessary, but ideally three milliliters should be collected to allow for repeat tests if indicated. Additionally, if ALA is to be measured, 0.5 mL of 30% glacial acetic acid must be added to the sample to obtain the correct acidity for measurement.^9^ Samples should be collected in a light-protected or amber plastic tube (Image) and subsequently frozen for transport. Freeze-thaw cycles should be avoided as these can result in decreased PBG concentrations.^10^ The turnaround time for commercial lab tests, which commonly use liquid chromatography-tandem mass spectrometry, ranges from 3–5 days.^4^,^9^,^10^

A fourfold increase from the reference ALA and PBG levels is used to identify several types of acute hepatic porphyria and further suspicion of others.^11^ Confirmatory and classification testing can use a variety of other means, including measurement of urine porphyrins, erythrocyte porphyrins, fecal porphyrins, and genetic analysis; however, these are best suited for the non-acute setting, commonly by specialists at porphyria centers.^4^,^10^,^11^

If urine PBG is elevated, or if a patient already has a confirmed diagnosis of porphyria, treatment should initiate in the ED. Precipitating or exacerbating factors should be withdrawn including environmental stressors, medications, or drops in caloric intake. Carbohydrate-loading prevents further upregulation of ALA.^11^ Symptomatic control should be obtained using only medications deemed “safe” for acute porphyria; one such database is readily available on the United Porphyrias Association website (www.porphyria.org).^12^ Hemin therapy, which reduces the production of hepatic porphyrin precursors, should be infused intravenously. Of importance, hemin treatment can be initiated at any stage during an acute attack. Recently, the US Food and Drug Administration approved givosiran, a small-interfering RNA (siRNA) that ultimately reduces the enzyme responsible for synthesizing ALA, as a new treatment option for a subset of patients with acute hepatic porphyria.^13^,^14^ However, this therapy is not yet readily available at many institutions given its significant cost compared to hemin.

A wide breadth of pathologies presents to the ED. Clinicians in this setting should consider acute porphyria in patients with neurovisceral symptoms and whose previous evaluations have been unrevealing. Many porphyria patients report years of ED visits before receiving an ultimate diagnosis, resulting in irreversible neurological damage and significant allocations of healthcare resources.^3^,^15^ Given that undiagnosed porphyria patients are often symptomatic when they seek acute care, the ED may be an ideal place to initiate competent screening.

## CONCLUSION

We present an unusual case in which acute porphyria was considered in the diagnosis of an ED patient with neurovisceral symptoms. Regrettably, the wrong screening test was ordered by the emergency physician resulting in a missed opportunity for identification. Given the high morbidity associated with acute porphyria, it is imperative that those who work in acute care settings be familiar with this collection of diseases and their screening and treatment.

## Figures and Tables

**Image:**
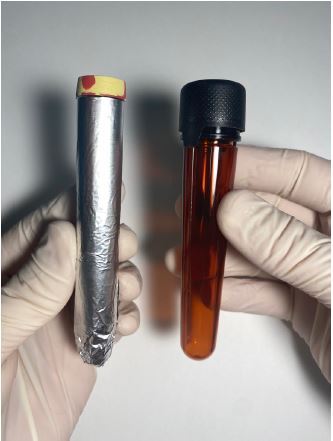
Appropriate sample tubes to collect and freeze urine samples for urine porphobilinogen assays. The amber tube (right) is ideal; however, if none can be obtained, a plain test tube may be covered in aluminum foil (left).
